# New proposal of silver diamine fluoride use in arresting approximal caries: study protocol for a randomized controlled trial

**DOI:** 10.1186/1745-6215-15-448

**Published:** 2014-11-19

**Authors:** Juliana Mattos-Silveira, Isabela Floriano, Fernanda R Ferreira, Maria E F Viganó, M A Frizzo, Alessandra Reyes, Tatiane F Novaes, Caroline M Moriyama, Daniela P Raggio, José C P Imparato, Fausto M Mendes, Mariana M Braga

**Affiliations:** Department of Pediatric Dentistry, School of Dentistry, University of São Paulo, Av. Lineu Prestes, 2227, São Paulo, SP 05508-000 Brazil

**Keywords:** Approximal surfaces, Dental caries, Flossing, Randomized clinical trials, Resin infiltration, Silver diamine fluoride

## Abstract

**Background:**

Approximal surfaces are a challenge to caries lesions control. Silver diamine fluoride (SDF) is a simple,low-cost and promisor intervention for arresting caries lesions, but it has never been tested on approximal surfaces. Our aim is to evaluate the efficacy and cost-efficacy of SDF in arresting initial lesions compared to resin infiltration and exclusively flossing (control group). Our second aim is to assess discomfort and satisfaction regarding interventions.

**Methods/design:**

This is a randomized clinical trial, double-blinded, placebo-controlled study. Children/adolescents presenting at least one approximal initial caries lesion in primary molars/permanent premolars and molars will be included. Surfaces with advanced dentine lesions identified by radiography and participants who refuse to participate or present negative behaviors will be excluded. A minimum sample size of 504 surfaces will be required for each subgroup. Individuals will be randomly allocated in three groups of interventions: SDF, resin infiltration, and control group. Depending on the allocation, the patients will receive the active treatment and respective placebo therapies. All patients will be oriented to daily flossing the included surfaces. Our primary outcome will be caries progression by clinical and radiographic examinations. Appointments will be timed and costs of materials will be considered to calculate cost-efficacy. Patient discomfort will be assessed after interventions. Parent and patient satisfaction with the treatment will be collected after treatment and in the last follow-up visit. Individuals will be assessed at 1 and 3 months after treatment to evaluate dental biofilm and at 6, 12, and 24 months to assess caries progression by visual examination and/or radiography. Multilevel analyses will be used to verify if the type of treatment influenced on the tested outcomes. Costs will be compared and analyses of cost-efficacy will be performed. Poisson analysis will test the association between intervention and reported discomfort and satisfaction.

**Discussion:**

Our hypothesis is that SDF is the most cost-efficacious option from all tested interventions. If our hypothesis is confirmed, the use of SDF in private and public contexts could represent an easier and effective option in the treatment of enamel approximal caries in children/adolescents.

**Trial registration:**

ClinicalTrials.gov (NCT01477385), Initial release: 11/16/2011: last update: 06/02/2014.

**Electronic supplementary material:**

The online version of this article (doi:10.1186/1745-6215-15-448) contains supplementary material, which is available to authorized users.

## Background

Approximal surfaces are a challenge regarding the control of caries lesions due to the area of contact between them and limited salivary access [1, 2]. In addition, poor compliance to flossing by children and adolescents [3] contributes to difficulty in arresting approximal caries lesions.

Despite flossing being the most suitable method for mechanical removal of biofilm from the interproximal area [4, 5], the controlling of approximal caries just by flossing has not been shown to be effective [6], probably because its use by children and adolescents is not constant and adequate [3]. Thus, early interventions to initial caries lesions become even more important to arrest these lesions and prevent cavitations or their progression into dentine.

Recently, the use of silver diamine fluoride (SDF), an effective cariostatic agent used in caries prevention since 1960 [7], has been proposed to arrest enamel caries lesions [8, 9]. A previous study of our group showed the SDF efficacy in arresting enamel caries lesions on occlusal surfaces of erupting permanent molars [8], which are areas of difficult mechanical control of biofilm, much like the approximal surfaces. However, this cariostatic agent has not been tested in the arrest of approximal caries lesions.

Other options, such as sealants and resin infiltration, have been pointed as promisor to controlling approximal caries lesions [10]. The resin infiltrant is a novel low-viscosity resin that promotes sealing into the lesion [11]. Previous studies have shown caries infiltration to be an efficacious treatment for permanent and primary teeth [12–15], superior to exclusively flossing [12, 13] and probably even approximal sealing [14].

Contrary to SDF, resin infiltration requires the use of a rubber dam and clamps. Thus, we hypothesized the resin infiltration would result in higher costs and reduce patient acceptance for arresting initial caries than the use of SDF. Therefore, not only the efficacy but also the cost-efficacy and patient discomfort/satisfaction should be comparatively investigated for these approaches, in order to propose a cost-effective and acceptable technique for controlling caries lesions on approximal surfaces.

The aim of this study is to evaluate the efficacy and cost-efficacy of SDF in arresting initial caries lesions compared to the resin infiltration and exclusively by flossing. Furthermore, patient discomfort, as well as parent and patient satisfaction with different treatments for initial caries lesions will be investigated.

## Methods/design

### Ethical considerations and registrations

The protocol was previously approved by the Research Ethics Committee of Dental School, University of São Paulo (protocol 140/11). Each child and adolescent must assent to participation in the study. Informed consents are obtained from their parents or guardians previous to their allocation in the study. This study protocol was also registered on ClinicalTrials.gov (NCT01477385 – November 16^th^, 2011) and written following CONSORT guidelines for randomized trials of non-pharmacologic treatment [16] and the Standard Protocol Items – Recommendations for Interventional Trials guidelines for clinical trial protocols.

### Study design

**Figure 1:**
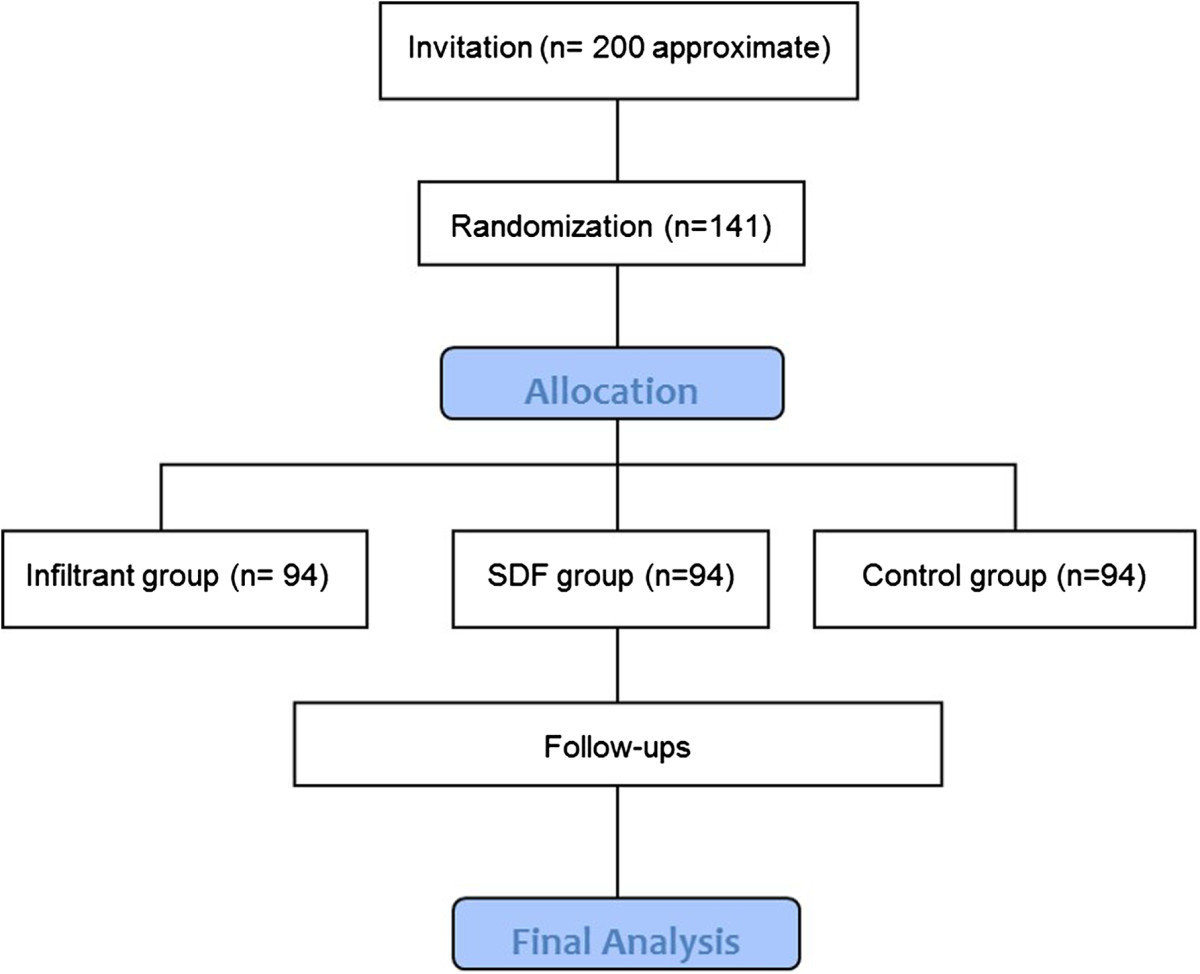
**CONSORT flow diagram of patient randomization.**

This is a randomized clinical trial, double-blinded, placebo-controlled study conducted in three-arm parallel groups. The participants will be allocated to one of the three arms in order to compare different options for arresting initial caries lesions (Figure 1). The participants and their parents/guardians, as well as the examiners, will not be aware of the patient’s allocation.

### Sample size calculation

To calculate the minimum sample size, we considered two possible comparisons: flossing vs. SDF/infiltration and SDF vs. infiltration. This approach follows since differences between SDF and infiltrant are expected to be lower and thus a bigger sample may be required to present statistical power. As no study has tested the SDF on approximal initial caries, we used differences between other tested treatments and/or similar treatments on other surfaces to estimate minimum expected difference among the groups tested in our protocol.

Considering flossing vs. other interventions, we considered a minimum difference of 30% [13], an alpha error of 5%, and a study power of 80%. A minimum sample size of 36 surfaces per group were found. To compare SDF and infiltrant, we considered 20% as minimum expected difference [9]. Then, the minimum sample size found was 86 surfaces. Rates considered for estimating differences among treatments were obtained from previous studies in permanent teeth. As caries progression tends to be faster in primary teeth [17], we believe similar or even smaller samples could achieve statistical power to prove existent differences in these teeth.

Considering we will include similar sample sizes for all groups, we assumed the biggest sample as reference (n = 258 surfaces). To ensure the power of the study, we added 30% to this sample to compensate possible follow-up drop-outs, totalizing a minimum sample size of 336 surfaces (112 surfaces/per group). Since child and adolescent patient characteristics were significantly different, we opted to conduct two parallel studies, with similar designs, each one including one of these groups of patients.

To compensate the clustering effect, since more than one surface may be included in each child/adolescent, we added an additional 50% on the calculation. Thus, we reached a final simple size of 504 surfaces (on average, 168 surfaces per group) to be included in this study.

### Participant selection

A total of 141 children (3 to 10 years old) and 141 adolescents (12 to 18 years old), who seek for dental treatment in our Dental School, will be evaluated according to inclusion/exclusion criteria as follows.

#### Inclusion criteria

Presence of at least one active initial caries lesion (scores 1, 2, or 3 according to International Caries Detection and Assessment System – ICDAS), detected by visual and tactile examination [18, 19] on approximal surfaces of primary molars for children or permanent premolar/molar for adolescents. For this purpose, surfaces will be evaluated after orthodontic rubber placement for at least 48 hours [20, 21], cleaned with dental flossing and clinically assessed by one trained and blinded examiner (IF, TFN, CMM) after rubber removal. The approximal surfaces of interest for the study will be those that presented full contact with the adjacent tooth. All the surfaces eligible for this study will be included.

#### Exclusion criteria

i) Presence of advanced dentine caries lesions detected by bitewing radiographic examinations (radiographic scores 4, 5, or 6) [15]. Only the surfaces presenting this condition will be excluded. The other eligible surfaces in the same patient will be maintained in the sample. ii) Children or adolescents that refuse to participate in the study or present negative behaviors.

Children that presented other needs for dental treatment will be referred to treatment in our Dental School. Moreover, all individuals will receive orientation about dietary and hygiene habits.

### Random allocation

The three parallel groups of interventions are defined as SDF, caries resin infiltration, and control group (exclusively flossing instructions).

The individuals will be randomly allocated to each group according to a sequence obtained in appropriate statistical software (Medcalc software version 12.4.0.0, Ostend, Belgium). The generated sequence will be distributed in opaque and sealed envelopes, which will be opened by the operators (JMS/FRF) only at the moment of the interventions.

All individuals enrolled in the study will receive in each included surface the active intervention, the placebo therapy, and instruction for daily flossing this specific surface. When the active treatment is exclusively flossing, two placebo therapies will be used, as described below. All procedures will be performed sequentially in a same surface by trained operators (JMS, FRF), in the same appointment.

### SDF group

#### Placebo therapy

Individuals will receive sterile water application, as a placebo, maintaining the same steps of resin infiltration technique. Absolute isolation will be simulated just positioning the rubber dam on teeth, without local infiltration anesthesia and clamp.

#### Intervention

Soft tissues will be protected with petroleum jelly to avoid staining and mucosal irritation, as recommended by the manufacturer. Moisture will be controlled by using cotton rolls and saliva ejectors. The adjacent tooth will be protected by plastic or a metal strip. SDF (Cariestop® 30% – Biodinâmica Química e Farmacêutica LTDA, Brasil) will be applied with a small disposable brush for 3 min. Then, the surface will be washed for 30s [22].

At the end, patients and their parents will receive instructions for daily flossing. Children will be oriented to flossing at least one time per day, flossing each approximal surface curving the floss around the base of teeth, holding it tautly between fingers, sliding it for entire surface and not provoking gingival traumatic injuries. Parents will also receive orientation about helping their children to floss. Despite instruction being given for flossing of all interproximal spaces, the operator stresses the need of flossing the specific areas presenting initial caries lesions.

### Resin infiltration group

#### Placebo therapy

Children will receive sterile water application, as a placebo, maintaining the same steps used for application of SDF.

#### Intervention

Local anesthesia and adaptation of the rubber dam and clamps will be performed. The adjacent tooth will be protected by plastic or metal strip. Hydrochloric acid 15% will be applied on the lesion for 120 s. Then, after washing and air-drying, the lesion will be dehydrated by applying 95% ethanol, followed by air-drying. The resin infiltrant will be applied on the lesion for 120 s and light-cured for 40s. Resin will be reapplied for 30s and light-cured again. All products used in resin infiltration are included in a specific kit commercialized by the manufacturers for this purpose (Icon® – Dental Milestones Guaranteed – DMG, Germany). At the end, patients and their parents will also receive instructions for daily flossing as described above.

### Control group

#### Placebo therapy

Children will receive sterile water application, as a placebo, maintaining the same steps of used for application of SDF and for resin infiltration, without local infiltration anesthesia and clamp.

The active treatment in this group will be only instruction for daily flossing as described above.

### Primary outcome

A single trained, calibrated, and blinded examiner (IF, TFN, CMM, AR) will assess caries lesions progression by direct visual exam using ICDAS and an adjunct index to assess caries lesion activity status [23]. The ICDAS score and the activity status will be assessed after tooth separation for at least 48 hours. The main outcome considered will be caries progression to evident dentine cavitated caries lesion. Bitewing radiographs will be taken to evaluate possible caries progression into dentin according to Ekstrand et al. [15]. The progression detected by radiographs or by ICDAS scores transition, as well as changes in the caries lesion activity status, will be considered as possible surrogate outcomes.

### Second outcome measurements

Patient discomfort will be assessed immediately after the sequence of treatments have been performed. Wong-Baker Faces Scale [24] will be applied to measure patient discomfort. The scale presents six figures from the happiest face to the unhappiest face in order to estimate discomfort level after procedure. An external examiner (MEFV/MFA) will instruct the child/adolescent to indicate which face represented his/her feeling regarding the procedures [20, 21].

We will also investigate parents’/guardians’ satisfaction regarding the patient’s treatment. The adolescent will be also asked to score his/her satisfaction. The assessments will be done in the first and last follow-up visits (1 and 24 months) and the examiners will ask them not to restrain from answering their actual opinion. Parents and adolescents should rank their satisfaction as: 0 – excellent; 1 – good; 2 – acceptable; 3 – bad [25].

Costs analyses will be performed based on the direct and indirect costs of the procedures. All appointments will be timed by an external examiner (MEFV/MFA). The number of expected and unexpected visits for each patient and the procedure performed in each session will be recorded, as well as their respective duration. To calculate the direct costs, the costs of materials used will be searched [26, 27]. These values will be inferences of market value obtained by a mean cost practiced by different dental stores. These data will be updated during the study. For the calculations, indirect costs will also be considered, as used in previous studies [26, 27].

Based on data collected for efficacy and cost analysis, we will propose a specific model for cost-efficacy estimation, based on previous published strategies [28].

### Independent variables

**Figure 2:**
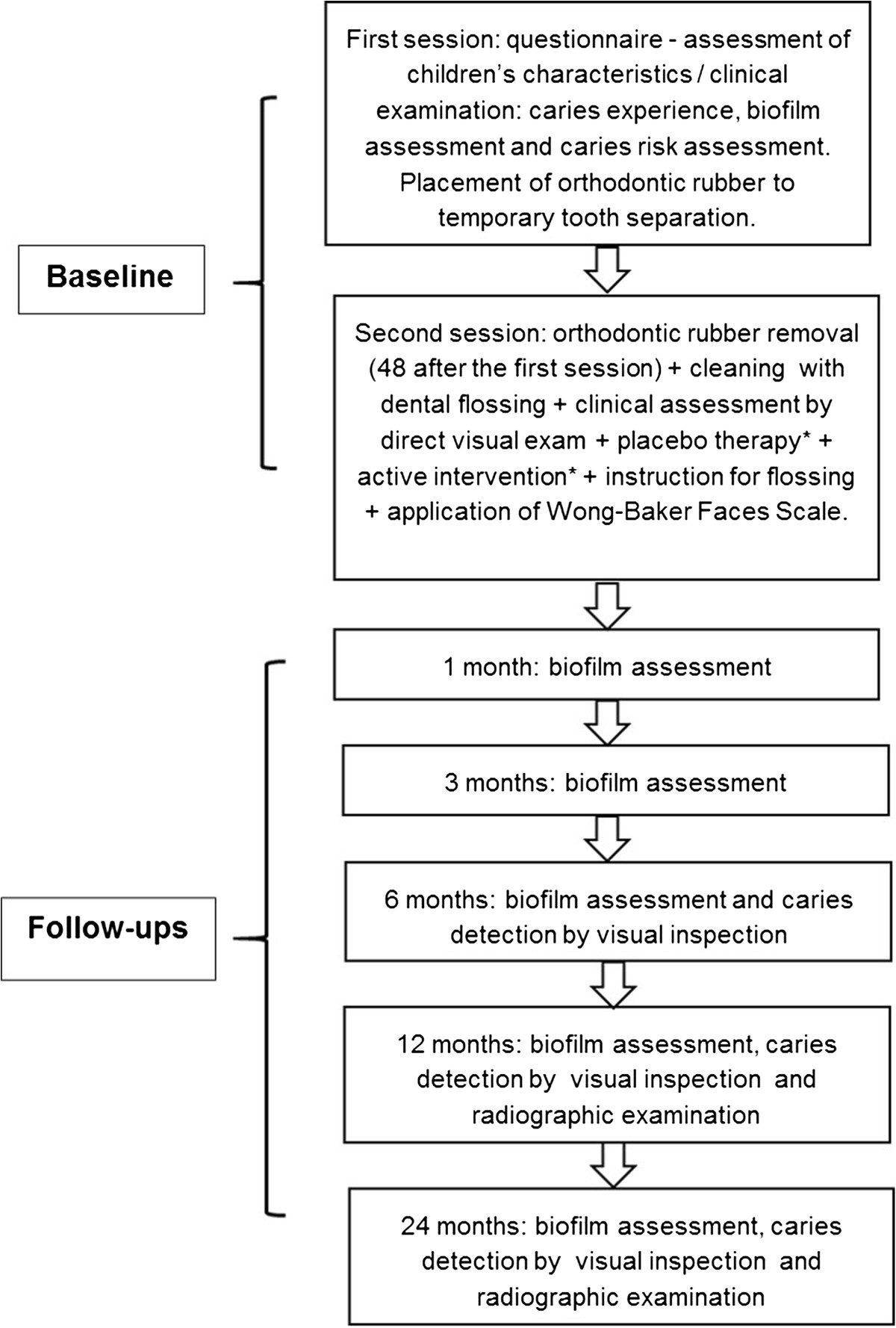
**Sequence of procedures performed for each recruited child**/**adolescent.** *Active and placebo therapies may be performed in different order depending on the group. In the control group, the active treatment is the flossing and two placebo therapies will be performed.

A structured questionnaire will be answered by the parents/guardians to check the individual characteristics of children/adolescents, such as socioeconomic, behavioral, medical, hygiene, use of fluoride, and dietary factors. One trained and calibrated examiner (JMS/FRF) will also assess caries experience using World Health Organization criteria (Decayed, Missing, Filled Surfaces in permanent teeth (DMFS) and DMFS in primary teeth (dmfs)) [29]; visible and disclosed biofilm [30, 31]; gingival papilla status of the approximal surfaces included [32]; and general biofilm by Simplified Oral Hygiene Index [33]. Individual caries risks will be evaluated by Cariogram Software [34] (Figure 2).

### Follow-up examinations

At baseline, independent variables will be collected for each patient. In addition, for each included surface, the ICDAS and radiographic scores will be registered, as well as the caries activity status.Patients will be assessed after 1, 3, 6, 12, and 24 months. At the 1- and 3-month follow-up the independent variables related to presence of biofilm will be re-evaluated. At other follow-ups (6, 12, and 24 months), direct visual examination of enrolled surfaces will be performed after tooth separation for 48 hours. Moreover, radiographic examination will be performed on the 12- and 24-month follow-up visits (Figure 2). The examinations will be performed as explained in previous sessions.

### Statistical analysis

The intra- and interexaminer reproducibilities in direct visual inspection and radiographic examination will be calculated by the unweighted and weighted Kappa test, depending on the evaluated outcome.

Univariate and multiple multilevel analyses will be used: i) to verify if the used treatment influences on efficacy and cost-efficacy for arresting initial caries lesions and ii) to test the association between different independent variables and treatment efficacy. Caries progression to evident cavities will be the main outcome considered in these cases. If necessary, surrogate outcomes will be used in these analyses. The relative risks and hazard ratios with 95% confidence interval (CI) will be calculated, respectively, after 2-year follow-up and considering all follow-up times. For all multilevel analyses, the surface, the tooth, and the child will be used as levels.

The following independent variables will be tested according to the level: i) surface: visible and disclosed biofilm on the approximal surface; ii) tooth: side; arch [35]; iii) child: age in years; number of daily toothbrushings; who toothbrushes; type of toothpaste; use of dental floss; who flosses; previous visit to a dentist; previous professional topical fluoride application; caries experience (dmfs + DMFS); simplified oral hygiene index [33]; number of active caries lesions in smooth surfaces; individual caries risk; and socioeconomic, behavioral, and dietary factors.

Poisson analysis will be used to assess the association between intervention and patient discomfort and parents’/adolescents’ satisfaction. The prevalence ratio or rate ratio with a 95% CI will be calculated, depending on the outcome considered. If necessary, relevant independent variables will be included for adjustment in the final models.

Interim-analyses will be performed on the primary outcome when 50% have completed 12-month and 24-month follow-up and also, when 100% of patients completed the 12-month follow-up.

The significance level for all analysis will be *P* < 0.05. For all multiples models tested, a level of significance of 0.20 will be considered for the variable entering into the model and a level of 0.05 for remaining in the model.

## Discussion

This trial aims to propose a possible efficacious treatment for initial caries lesion on approximal surfaces, which could be useful in private and public contexts representing an easier and effective option to treatment of enamel approximal caries in children and adolescents.

In theory, it is known that initial caries lesions could be arrested by biofilm removal [36]. In fact, dental flossing would be the simplest procedure for biofilm removal to control initial lesions on approximal surfaces. However, this control depends on correct and frequent flossing by patients [3], which could explain the difficulty in arresting lesions in these specifics areas [6]. When flossing is not effective, it is necessary to resort to other possibilities to control caries lesions. That is why it is important to evaluate flossing comparatively to other available options in order to seek for the simplest and most cost-efficacious/effective treatment.

Previous studies evaluated the preventive effect of SDF [22] and/or its efficacy on dentine lesions [7, 37]. However, few studies assessed its efficacy on initial caries lesion [8, 9]. In addition, no previous study verified its application on approximal surfaces. A systematic review showed that SDF seems to be more effective than fluoride varnish [38]. A possible explanation for this superior effect could be the fluoride concentration on SDF, which is higher than that found in the fluoride varnish [39]. However, the effect of SDF on caries lesions could be explained not only by fluoride action, but also by the silver ions, which react with thiol groups in amino and nucleic acids leading to bacterial lysis [38], and thus to probable quicker caries arrestment [8, 40], especially if compared to other options of arresting initial caries lesions such as other fluorides.

There are some aesthetic concerns regarding the use of SDF since it could cause black staining on carious lesions due to the precipitation of silver [38]. However, aesthetics did not seem to be jeopardized in the case of initial caries lesions on approximal surfaces of posterior teeth due to their localization. However, the staining could be a limitation of our study regarding examiners’ blinding, since it could suggest which surfaces were treated with SDF. Nevertheless, inactive lesions could also present dark coloring, minimizing this limitation [8, 22].

On the other hand, resin infiltration is a new procedure, with promising results, but it could be costly and be less accepted by patients. Previous studies have shown that flossing is less effective than infiltration, sealing, and other fluoride treatments [13, 14, 41, 42]. To the best of our knowledge, there is no study that has previously compared flossing, which would be the simplest technique to be used on approximal surfaces, with SDF. As children/adolescents who will be enrolled in this study live in an area of fluoridated water and regularly use fluoridated toothpaste, if we had not included the control group, we could not affirm which is the actual simplest and most cost-efficacious technique for approximal initial caries, which is why we decided to conduct this study in three-arm parallel groups rather than design a non-inferiority trial comparing just SDF with resin infiltration. However, depending on the results of this study a posterior effectiveness trial could be conducted. Besides, all children have been exposed to daily fluoride, have been motivated to floss every day, and are monitored regularly. Therefore, we believe that using flossing as a control group has not compromised the ethics of the trial.

To compare these three interventions, we considered important the use of placebo therapies to ensure that interventions are as similar as possible among individuals. If individuals are aware of which group they will be allocated to, they may judge more complex techniques, which demands a lot of clinical steps, as more effective and underestimate the correct daily flossing in these cases. Due to the vulnerable individuals [43] included in our study, the perfect placebo therapies could be questionable. Thus, for ethical issues, the placebo of resin infiltration will be performed just simulating the rubber dam on teeth, without local infiltration anesthesia and clamp. Despite being an imperfect placebo, simple measures like this can be enough to blind patient regarding adopted treatment [44]. Therefore, in such a type of imperfect placebo-controlled study, we expect that daily flossing is not influenced by the intervention received.

On the other hand, we must consider that sequential treatments may influence patients’ perception about the treatment. Because of its sensitivity to light, a light-curing or light curing simulation will have to be applied after the use of SDF. Thus, the sequence has been standardized and our option was to apply the faces scale at the end of all procedures, and not only after the active treatment, despite the latter being ideal. The use of the scale only after active treatment would compromise the blinding, which would be worse. In addition, using the procedures in sequence will make the sessions longer. Therefore, we believe we should discuss our results considering that a session for application of SDF on initial approximal caries lesions would certainly consume less time than will be presented in the results of this trial. A pilot study is ongoing to check the partial times for each approach separately and the influence of each individual treatment and their respective placebos on patient perception.

Finally, we should expect a degree of memory bias, especially a named choice-supportive bias [45] when we checking daily flossing. Actually, children and adolescents, as well their parents/guardians, may be conscious of the correct flossing behavior [46], especially in a study like ours, since they are orientated to act in a specific way during a certain time. Therefore, they could report the daily use of dental floss, regardless of this being the case. This bias could underestimate the actual efficacy of this practice and should be considered when interpreting our findings.

At the end of this study we expect to contribute to point out the option(s) which would be definitely more efficacious/cost-effective than the mechanical removal of biofilm from approximal surface by flossing.

## Trial status

This is an ongoing trial, which is still recruiting the patients at this moment.

## Authors’ original submitted files for images

Below are the links to the authors’ original submitted files for images. Authors’ original file for figure 1 Authors’ original file for figure 2

## Abbreviations

CI: Confidence interval
DMFS: Decayed, missing, filled surfaces in permanent teeth
Dmfs: Decayed, missing, filled surfaces in primary teeth
ICDAS: International Caries Detection and Assessment System
SDF: Silver diamine fluoride.

## References

[1] Cagetti MG, Campus G, Sale S, Cocco F, Strohmenger L, Lingström P (2011). Association between interdental plaque acidogenicity and caries risk at surface level: a cross sectional study in primary dentition. Int J Paediatr Dent.

[2] Seki M, Karakama F, Terajima T, Ichikawa Y, Ozaki T, Yoshida S, Yamashita Y (2003). Evaluation of mutans streptococci in plaque and saliva: correlation with caries development in preschool children. J Dent.

[3] Ashkenazi M, Bidoosi M, Levin L (2012). Factors associated with reduced compliance of children to dental preventive measures. Odontology.

[4] Corby PM, Biesbrock A, Bartizek R, Corby AL, Monteverde R, Ceschin R, Bretz WA (2008). Treatment outcomes of dental flossing in twins: molecular analysis of the interproximal microflora. J Periodontol.

[5] Merchant AT (2009). Flossing for 2 weeks reduces microbes associated with oral disease. J Evid Based Dent Pract.

[6] Hujoel PP, Cunha-Cruz J, Banting DW, Loesche WJ (2006). Dental flossing and interproximal caries: a systematic review. J Dent Res.

[7] Chu CH, Lo EC, Lin HC (2002). Effectiveness of silver diamine fluoride and sodium fluoride varnish in arresting dentin caries in Chinese pre-school children. J Dent Res.

[8] Braga MM, Mendes FM, De Benedetto MS, Imparato JC (2009). Effect of silver diammine fluoride on incipient caries lesions in erupting permanent first molars: a pilot study. J Dent Child.

[9] Liu BY, Lo EC, Chu CH, Lin HC (2012). Randomized trial on fluorides and sealants for fissure caries prevention. J Dent Res.

[10] Tellez M, Gomez J, Kaur S, Pretty IA, Ellwood R, Ismail AI (2013). Non-surgical management methods of noncavitated carious lesions. Community Dent Oral Epidemiol.

[11] Phark JH, Duarte S, Meyer-Lueckel H, Paris S (2009). Caries infiltration with resins: a novel treatment option for interproximal caries. Compend Contin Educ Dent.

[12] Meyer-Lueckel H, Bitter K, Paris S (2012). Randomized controlled clinical trial on proximal caries infiltration: three-year follow-up. Caries Res.

[13] Paris S, Hopfenmuller W, Meyer-Lueckel H (2010). Resin infiltration of caries lesions: an efficacy randomized trial. J Dent Res.

[14] Martignon S, Ekstrand KR, Gomez J, Lara JS, Cortes A (2012). Infiltrating/sealing proximal caries lesions: a 3-year randomized clinical trial. J Dent Res.

[15] Ekstrand KR, Bakhshandeh A, Martignon S (2010). Treatment of proximal superficial caries lesions on primary molar teeth with resin infiltration and fluoride varnish versus fluoride varnish only: efficacy after 1 year. Caries Res.

[16] Boutron I, Moher D, Altman DG, Schulz KF, Ravaud P, Group C (2008). Methods and processes of the CONSORT Group: example of an extension for trials assessing nonpharmacologic treatments. Ann Intern Med.

[17] Sonju Clasen AB, Ogaard B, Duschner H, Ruben J, Arends J, Sonju T (1997). Caries development in fluoridated and non-fluoridated deciduous and permanent enamel in situ examined by microradiography and confocal laser scanning microscopy. Adv Dent Res.

[18] Braga MM, Mendes FM, Ekstrand KR (2010). Detection activity assessment and diagnosis of dental caries lesions. Dent Clin North Am.

[19] Ismail AI, Sohn W, Tellez M, Amaya A, Sen A, Hasson H, Pitts NB (2007). The International Caries Detection and Assessment System (ICDAS): an integrated system for measuring dental caries. Community Dent Oral Epidemiol.

[20] Novaes TF, Matos R, Raggio DP, Imparato JC, Braga MM, Mendes FM (2010). Influence of the discomfort reported by children on the performance of approximal caries detection methods. Caries Res.

[21] Novaes TF, Matos R, Raggio DP, Braga MM, Mendes FM (2012). Children’s discomfort in assessments using different methods for approximal caries detection. Braz Oral Res.

[22] Llodra JC, Rodriguez A, Ferrer B, Menardia V, Ramos T, Morato M (2005). Efficacy of silver diamine fluoride for caries reduction in primary teeth and first permanent molars of schoolchildren: 36-month clinical trial. J Dent Res.

[23] ICDAS-Committee (2005). International Caries Detection and Assessment System (ICDAS II) Criteria Manual. Workshop held in Baltimore, Maryland, March 12th-14th 2005.

[24] Wong DL, Baker CM (1988). Pain in children: comparison of assessment scales. Pediatr Nurs.

[25] Hämmerle CH, Jung RE, Sanz M, Chen S, Martin WC, Jackowski J, Multicenter study group (2012). Submerged and transmucosal healing yield the same clinical outcomes with two-piece implants in the anterior maxilla and mandible: interim 1-year results of a randomized, controlled clinical trial. Clin Oral Implants Res.

[26] Takanashi Y, Penrod JR, Lund JP, Feine JS (2004). A cost comparison of mandibular two-implant overdenture and conventional denture treatment. Int J Prosthodont.

[27] Kawai Y, Murakami H, Takanashi Y, Lund JP, Feine JS (2010). Efficient resource use in simplified complete denture fabrication. J Prosthodont.

[28] Stevenson M, Lloyd-Jones M, Morgan MY, Wong R (2012). Non-invasive diagnostic assessment tools for the detection of liver fibrosis in patients with suspected alcohol-related liver disease: a systematic review and economic evaluation. Health Technol Assess.

[29] WHO (1997). Oral health surveys: basic methods.

[30] Silness J, Loe H (1964). Periodontal disease in pregnancy. ii. Correlation between oral hygiene and periodontal condition. Acta Odontol Scand.

[31] Block PL, Lobene RR, Derdivanis JP (1972). A two-tone dye test for dental plaque. J Periodontol.

[32] Ekstrand KR, Bruun G, Bruun M (1998). Plaque and gingival status as indicators for caries progression on approximal surfaces. Caries Res.

[33] Greene JC, Vermillion JR (1964). The simplified oral hygiene index. J Am Dent Assoc.

[34] Bratthall D, Hänsel Petersson G (2005). Cariogram – a multifactorial risk assessment model for a multifactorial disease. Community Dent Oral Epidemiol.

[35] Ekstrand KR, Christiansen J, Christiansen ME (2003). Time and duration of eruption of first and second permanent molars: a longitudinal investigation. Community Dent Oral Epidemiol.

[36] Zero DT, Fontana M, Martinez-Mier EA, Ferreira-Zandona A, Ando M, Gonzalez-Cabezas C, Bayne S (2009). The biology, prevention, diagnosis and treatment of dental caries: scientific advances in the United States. J Am Dent Assoc.

[37] Lo EC, Chu CH, Lin HC (2001). A community-based caries control program for pre-school children using topical fluorides: 18-month results. J Dent Res.

[38] Rosenblatt A, Stamford TC, Niederman R (2009). Silver diamine fluoride: a caries “silver-fluoride bullet”. J Dent Res.

[39] Mei ML, Chu CH, Lo EC, Samaranayake LP (2013). Fluoride and silver concentrations of silver diammine fluoride solutions for dental use. Int J Paediatr Dent.

[40] Chu CH, Lo EC (2008). Promoting caries arrest in children with silver diamine fluoride: a review. Oral Health Prev Dent.

[41] Peyron M, Matsson L, Birkhed D (1992). Progression of approximal caries in primary molars and the effect of Duraphat treatment. Scand J Dent Res.

[42] Jiang H, Bian Z, Tai BJ, Du MQ, Peng B (2005). The effect of a bi-annual professional application of APF foam on dental caries increment in primary teeth: 24-month clinical trial. J Dent Res.

[43] De Benedetto MS, Morais CC, Novaes TF, de Almeida RJ, Braga MM, Mendes FM (2011). Comparing the reliability of a new fluorescence camera with conventional laser fluorescence devices in detecting caries lesions in occlusal and smooth surfaces of primary teeth. Lasers Med Sci.

[44] Fahy C, Costi D, Cyna A (2011). Invasive 'placebo’ controls: have we lost sight of whom we are blinding?. Paediatr Anaesth.

[45] Mather M, Shafir E, Johnson MK (2003). Remembering chosen and assigned options. Mem Cogn.

[46] Ashkenazi M, Cohen R, Levin L (2007). Self-reported compliance with preventive measures among regularly attending pediatric patients. J Dent Educ.

