# Supplementary figures and images for: New proposal of silver diamine fluoride use in arresting approximal caries: study protocol for a randomized controlled trial
Source: Trials. 2014 Nov 19;15:448. doi: 10.1186/1745-6215-15-448 (PMC4255679; doi:10.1186/1745-6215-15-448)

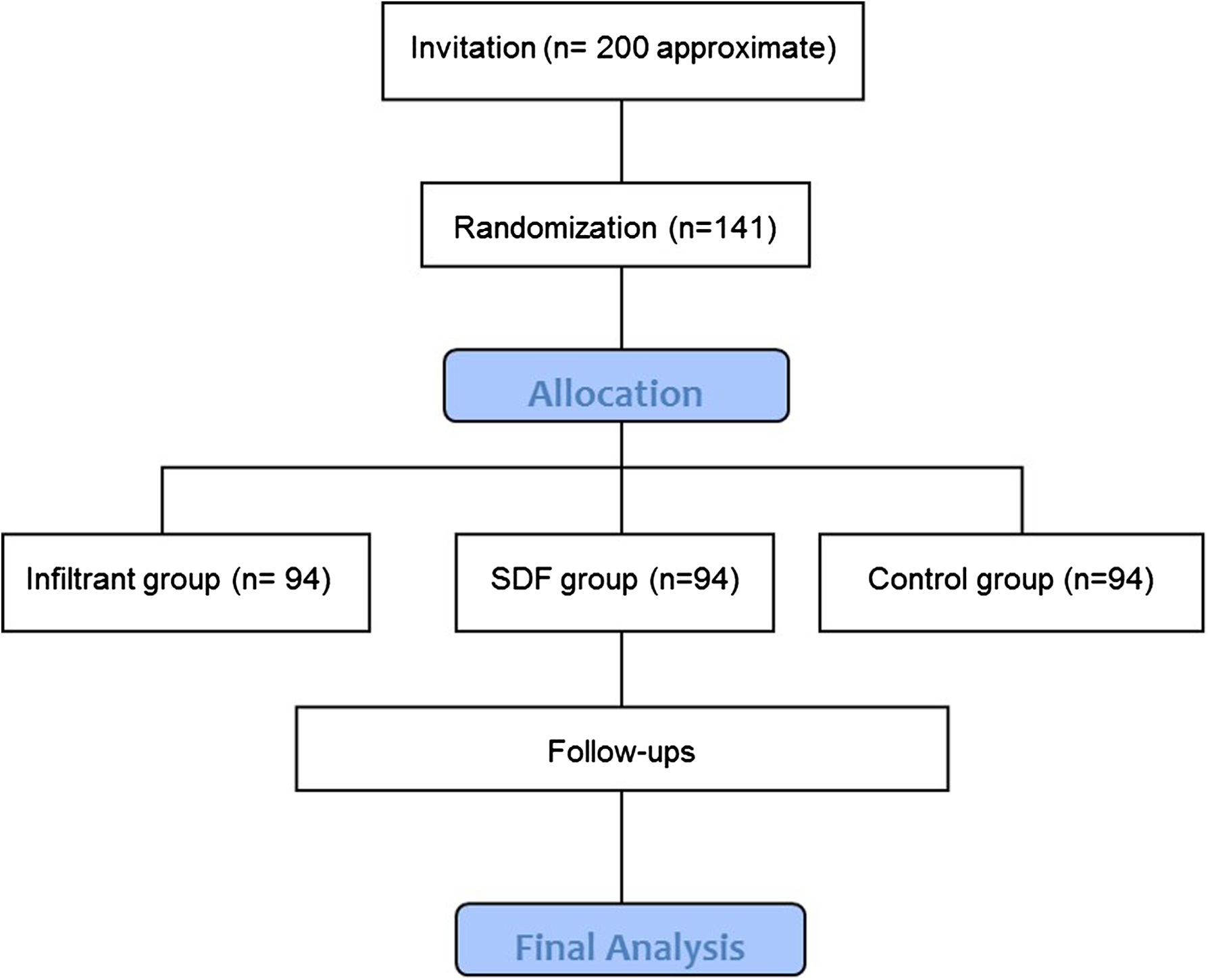

Supplement: Supplementary file 1 — Authors’ original file for figure 1 [file 13063_2014_2324_MOESM1_ESM.tif]

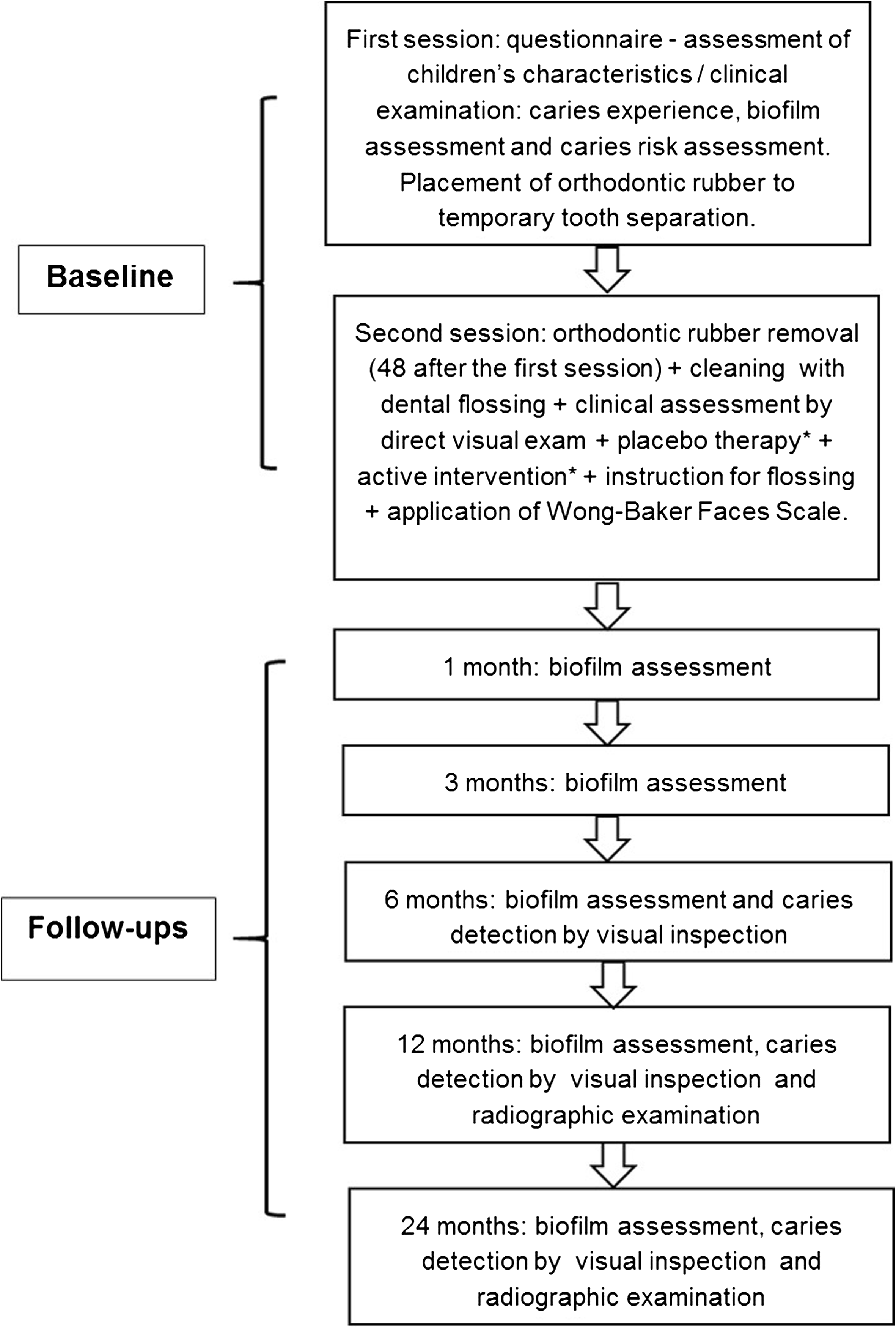

Supplement: Supplementary file 2 — Authors’ original file for figure 2 [file 13063_2014_2324_MOESM2_ESM.tif]
